# *Enpp2* haploinsufficiency induces an eye-open-at-birth phenotype in the DBA/2 background

**DOI:** 10.17912/micropub.biology.001212

**Published:** 2024-04-24

**Authors:** Seiichi Koike, Kazuko Keino-Masu, Masayuki Masu

**Affiliations:** 1 Graduate School of Comprehensive Human Sciences, University of Tsukuba, Tsukuba, Ibaraki, Japan; 2 Department of Molecular Neurobiology, Institute of Medicine, University of Tsukuba, Tsukuba, Ibaraki, Japan; 3 Engineering for Research, University of Toyama, Toyama, Toyama, Japan

## Abstract

Autotaxin, encoded by the *Enpp2* gene, produces lysophosphatidic acid (LPA), which exerts numerous biological functions via its cognate receptors. *Enpp2* null mutant mice die by embryonic day 9.5 owing to aberrant vascular development in the yolk sac, preventing analysis after that period. In this study, we found that *Enpp2* heterozygous mice in the DBA/2 genetic background showed an eye-open-at-birth phenotype at high frequency, caused by failure of eyelid closure during the embryonic stage. Notably, wildtype pups from the *Enpp2* heterozygous dam showed the phenotype, although at lower frequency, suggesting that maternal LPA affects the embryonic development.

**Figure 1.**
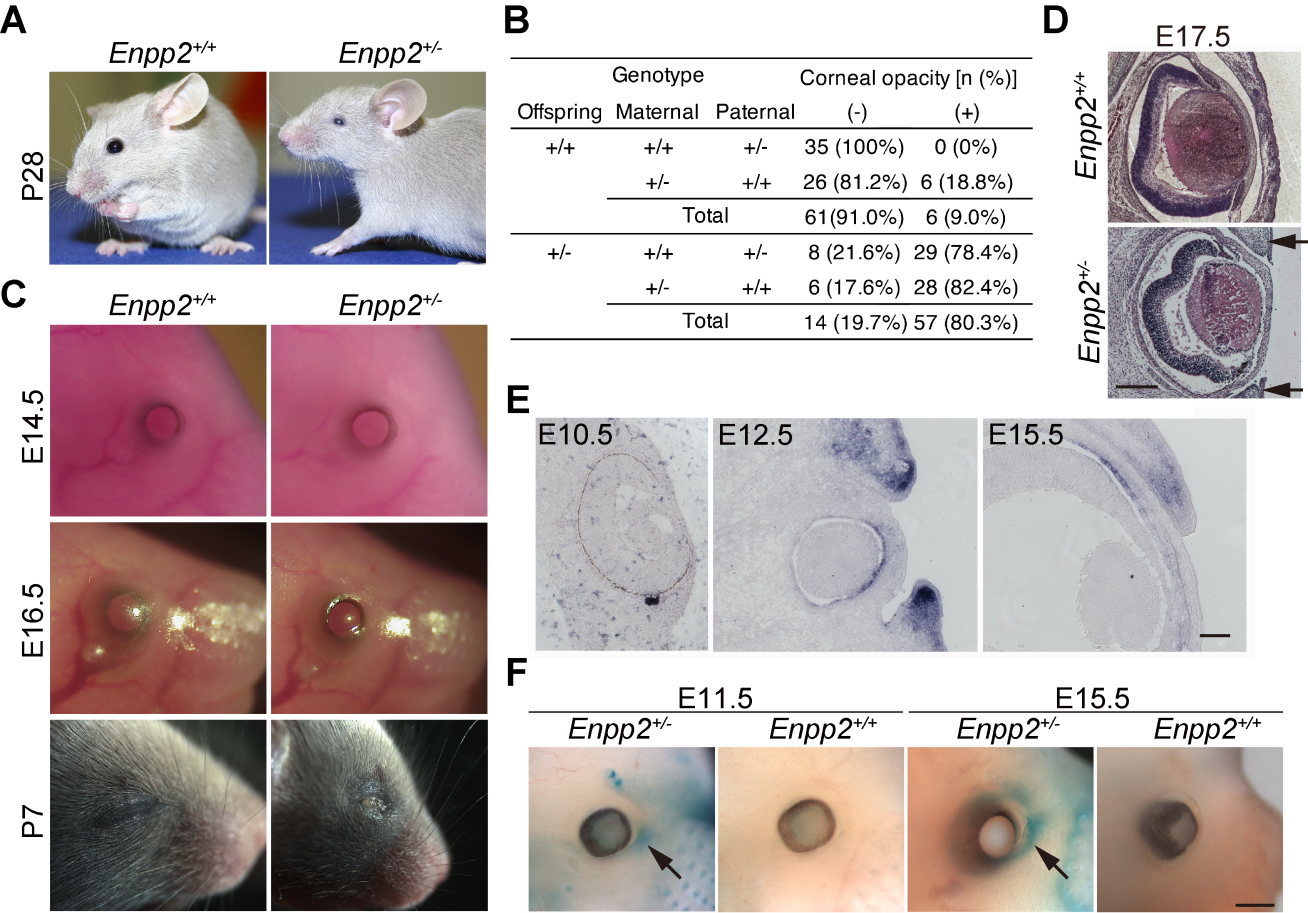
Eye-open-at-birth phenotype of *Enpp2* ^+/-^ mice in the DBA2 background. **(A)** Eye appearance of *Enpp2 ^+/+^* or *Enpp2 ^+/-^* mice at P28. About 80% of the *Enpp2 ^+/-^* mice showed smaller eyes and corneal opacity after 3 consecutive backcrosses to DBA/2J. **(B)** Table showing the numbers and percentages of mice with corneal opacity at P28, after 4 consecutive backcrosses to DBA/2J. The numbers and percentages of the offsprings with EOB are classified and shown according to the parental genotypes. **(C)** Eye-open phenotype of *Enpp2 ^+/-^* embryos and neonates. The eyelids were open in both the *Enpp2 ^+/+^* and the *Enpp2 ^+/-^* embryos at E14.5. In the *Enpp2 ^+/+^* embryos, the eyelids were closed at E16.5 and remained closed at P7, whereas in the *Enpp2 ^+/-^* embryos, the eyelids remained open throughout the embryonic and neonetal periods. **(D)** Hematoxylin-eosin-stained transverse slices of the eyes of *Enpp2 ^+/+^* or *Enpp2 ^+/-^* embryos at E17.5. Eyelid epithelial extension was impaired in the *Enpp2 ^+/-^* embryos (black arrows). Scale bar, 300 µm. **(E)** *In situ* hybridization of *Ennp2* . At E10.5, no signal was observed. At E12.5, when the eyelids were extended, strong *Enpp2* expression was observed in the mesenchymal cells just beneath the extending epithelium, which persisted until E15.5. Scale bar, 50 µm. **(F)** LacZ staining of *Enpp2 ^+/+^* or *Enpp2 ^+/-^* embryos. At E11.5, the signals were detected in the eyelids and whisker hair follicles of the *Enpp2 ^+/-^* embryos. The signals in the eyelid region remained strong until E15.5. The signals were not observed in the *Enpp2 ^+/+^* embryo. Scale bar, 400 µm.

## Description

Autotaxin, also known as ectonucleotide pyrophosphatase/phosphodiesterase 2 (Enpp2), is an ectoenzyme responsible for the production of lysophosphatidic acid (LPA) in the extracellular space (Kano et al., 2022; Perrakis and Moolenaar, 2014; Stefan et al., 2005; Tokumura et al., 2002; Umezu-Goto et al., 2002) . LPA is a lipid mediator possessing a wide variety of biological functions, including cell proliferation, migration, and survival (Kano et al., 2022; Luquain et al., 2003) . These effects are mediated by its G protein-coupled receptors (LPA _1–6_ ), which activate intracellular signal pathways (Kano et al., 2022; Yung et al., 2014) . *Enpp2* ^-/-^ mice exhibit embryonic lethality at embryonic day 9.5 (E9.5) owing to angiogenic defects in the yolk sac (Koike et al., 2009; Tanaka et al., 2006; van Meeteren et al., 2006) . It is thus impossible to investigate the functions of autotaxin after this time period using conventional knockout (KO) mice. To overcome this difficulty, conditional KO mice or transgenic mice were used to examine autotaxin functions in the adult, including inflammation, fibrosis, and obesity (Dusaulcy et al., 2011; Nikitopoulou et al., 2022; Oikonomou et al., 2012) . Additionally, functional analysis has been performed using heterozygous mice, in which the LPA level in the circulation was reduced to approximately 50% of the level of that in wildtype (WT) mice (Tanaka et al., 2006; van Meeteren et al., 2006) : platelet-dependent thrombus formation, suppression of adipocyte hyperplasia, and impaired insulin signaling were reported in *Enpp2* heterozygous mice (D’Souza et al., 2018; Nishimura et al., 2014; Pamuklar et al., 2009).

In this study, we examined the impact of genetic background on the *Enpp2* mutation because in general, differences in genetic background can weaken or strengthen the phenotypes of KO mice (Montagutelli, 2000) . We backcrossed *Enpp2* heterozygous mice, maintained on a mixed genetic background of C57BL/6N and 129SvJ, to various inbred strains, including C57BL/6N, 129SvJ, BALB/cA, C3H/HeJ, and DBA/2J. After backcrosses to DBA/2J for 3 consecutive generations, we noticed for the first time that some postnatal mice developed corneal opacity ( Fig. 1A ): none of the mice backcrossed to other inbred strains developed such a phenotype nor showed other visible changes. The frequency of corneal opacity at postnatal day 28 (P28) was about 80% in the *Enpp2 ^+/-^* mice in the offspring backcrossed to DBA/2J for 4 consecutive generations ( Fig. 1B ). Intriguingly, a small number (8.9%) of the WT mice also showed a similar phenotype ( Fig. 1B ). To eliminate the possibility that a spontaneous mutation affecting eye development occurred during backcrossing, we conducted an independent backcross to DBA/2J and found that the same phenotype appeared at a similar frequency. When examined separately by each parent’s genotype, the WT pups with corneal opacity were observed in those born from *Enpp2 ^+/-^* females mated with WT males, but not in those born from WT females mated with *Enpp2 ^+/-^* males ( Fig. 1B ). These data imply that the maternal genotype influences the phenotypic appearance of the offspring.

Previous studies have demonstrated a correlation between corneal opacity and an eye-open-at-birth (EOB) phenotype. In mice, eyelid development begins with the groove extending over the cornea around E12, and the eyelid primordia meet and fuse around E16.5 (Findlater et al., 1993) . The eyelids remain fused until P12-14 when they reopen as a result of apoptosis of the eyelid tissues. When the eyelids are not closed at the embryonic stage, corneal injury induces inflammation and opacity after birh (Nunomura et al., 2021; Wu et al., 2016) . To determine whether the ocular abnormalities observed in *Enpp2* ^+/-^ mice are caused by EOB, we examined the eye development of embryos generated by mating WT female mice with *Enpp2 ^+/-^* male mice to minimize maternal effects. The eyelid primordia were formed in all the embryos at E12.5 to E14.5 ( Fig. 1C ). At E16.5, the eyelids had already fused in the WT embryos, but they remained open in the *Enpp2 ^+/-^* embryos ( Fig. 1C ). At P7, the eyelids remained closed in the WT mice, whereas the *Enpp2 ^+/-^* mice showed opened eyelids and corneal opacity ( Fig. 1C ). Histological analysis of transverse slices of the eye at E17.5 revealed that the epithelial sheets over the eye were not formed in the *Enpp2 ^+/-^* embryos, whereas no apparent abnormalities were observed in the lens, retina, or cornea ( Fig. 1D ). These data indicate that ocular abnormalities observed in postnatal *Enpp2 ^+/-^* mice resulted from a failure of eyelid extension and closure during the embryonic stage. The corneal opacity seems to be a secondary effect resulting from the injury caused by the absence of eyelids and loss of physical protection.

Finally, we examined the *Enpp2* expression in the eye region of the embryos. Initially, we detected *Enpp2* mRNA using *in situ* hybridizaton. At E10.5, *Enpp2* signals were undetectable around the eye ( Fig. 1E ). At E12.5, strong signals were detected in the mesenchymal cells, especially beneath the extending epithelium, but not in the epithelial cells of the eyelids. The expression remained detectable until E15.5. Next, we investigated *Enpp2* expression by means of wholemount LacZ staining, as the *LacZ* gene was knocked in the *Enpp2* KO allele. The signal was weakly detected around the eye at E11.5 and and had become stronger at E15.5 ( Fig. 1F ). The signals were not detected in the WT mice, suggesting the specificity of the lacZ reaction ( Fig. 1F ).

Our data indicate that *Enpp2* is expressed in the mesenchymal cells of eyelids when the eyelids extend and that decreased *Enpp2* expression induces the EOB phenotype in the DBA/2J background, suggesting that *Enpp2* is required for the proliferation and/or migration of mesenchymal cells in the eyelids. Why the EOB phenotype appears only in the DBA/2J background is unknown. However, given that DBA/2J is a model for congenital experimental glaucoma (Turner et al., 2017) , the strain may have unidentified susceptibility to eye diseases associated with maldevelopment of the anterior eye segment. Interestingly, heterozygosity of the dam partially induces the EOB phenotype in their WT offspring, suggesting that LPA in the dam somehow affects the eye development of their embryos. Although it also remains unknown whether LPA is supplied from the matermal circulation to the embryo, it is possible that an unidentified LPA transporter in the placenta facilitates transport of LPA as amino acids and fatty acids are transported (Brett et al., 2014) and extracellular LPA can activate peroxisome proliferator-activated receptor (PPARγ) in monocytic cells intracellularly across cell membranes (McIntyre et al., 2003) . Investigating how maternal LPA impacts embryonic development will be necessary in future.

## Methods

**Animals**

All experimental procedures involving animals were approved by the Animal Care and Use Committee of the University of Tsukuba and performed in accordance with its guidelines. *Enpp2* KO mice (B6;129SvJ- *Enpp2 ^tm1Mmas^* ) were generated by use of homologous recombination in 129/Ola-derived ES cells (Koike et al., 2009) . The mutant mice were backcrossed to C57BL/6N, 129SvJ, BALB/cA, C3H/HeJ, and DBA/2J, which were purchased from CLEA Japan. Offspring were genotyped by use of PCR using the primers 5’-CTGCTGAAACTTAATGCACTGGAC-3’ ( *Enpp2* forward), 5’-TGTGTAAGTCAGGGAACAACTCTG-3’ ( *Enpp2* reverse), and 5’-TGCTCCAGACTGCCTTGGGAAAAG-3’ ( *neo* ). Noon of the day when a vaginal plug was observed was taken as embryonic day 0.5 (E0.5). Embryos were taken after mice were sacrificed by means of cervical dislocation.

***In situ* hybridization**

C57BL/6N embryos were fixed with 4% paraformaldehyde (PFA) in phosphate-buffered saline (PBS). After the brains were incubated in 30% sucrose/PBS at 4°C overnight and embedded in OCT compound (Sakura Finetek Japan), 10-µm-thick slices were cut by use of a cryostat CM1850 (Leica). The slices were treated with 1 µg/mL proteinase K in PBS with 0.1% Tween-20 (PBT) at 37°C for 5 min, washed and fixed with 4% PFA, and hybridized with 1 µg/mL digoxigenin (DIG)-labeled antisense RNA probe (nt 678–1323 of mouse *Enpp2* cDNA; GenBank accession number NM015744 ) in a hybridization solution (50% formamide, 5× SSC pH 4.5, 1% SDS, 50 µg/mL heparin, 50 µg/mL yeast RNA) at 65°C for 16 h. The slides were washed with 50% formamide, 5× SSC, 1% SDS at 65°C for 30 min, and with 50% formamide, 2× SSC at 65°C for 30 min 3 times, and then incubated with an alkaline phosphatase-conjugated anti-DIG antibody (Sigma-Aldrich) at 4°C overnight. After washing with Tris-buffered saline with 0.1% Tween-20, signals were detected by use of BM purple (Sigma-Aldrich) in the presence of 2 mM levamisole (Sigma-Aldrich) at room temperature for 1–5 days.

**LacZ staining**

*Enpp2 ^+/+^* or *Enpp2 ^+/-^* embryos were fixed with 2% PFA and 0.2% glutaraldehyde in PBS. After rinsing with PBS 3 times, LacZ staining was carried out by incubation of the embryos in PBS containing 5 mM K _4_ Fe(CN) _6_ , 5 mM K _3_ Fe(CN) _6_ , 1 mg/ml X-gal, 2 mM MgCl _2_ , 0.02% Nonidet P40, and 0.01% Na deoxycholate.

**Histology**

For histological examination, mouse embryos were fixed with 4% PFA in PBS. Subsequently, the fixed embryos were processed and embedded in paraffin by use of a tissue processor ASP200 (Leica). Slices of 4-µm thickness were then cut with a microtome RM2145 (Leica) and stained with hematoxylin-eosin (Muto Pure Chemicals).
